# Toxic exposure and kidney function: Urinary creatinine and glomerular filtration rate in traditional sulfur miners

**DOI:** 10.1016/j.toxrep.2026.102266

**Published:** 2026-04-29

**Authors:** Septian Hadi Susetyo, Azham Umar Abidin, Taiki Nagaya, Emi Kawaguchi, Yasuto Matsui

**Affiliations:** a Department of Environmental Engineering, Faculty of Civil and Environmental Engineering, Institut Teknologi Bandung (ITB), Indonesia; b Department of Environmental Engineering, Faculty of Civil Engineering and Planning, Universitas Islam Indonesia, Indonesia; c Laboratory of Safety and Occupational Health Engineering (Agency for Health, Safety and Environment) laboratory, Kyoto University, Kyoto 606-8501, Japan

**Keywords:** Toxic natural pollution, Sulfur mining, Urine creatinine, Kidney function, Glomerular filtration rate

## Abstract

The Ijen Crater Volcano, recognized by UNESCO as a biosphere reserve, has a high sulfur content and is the main source of traditional sulfur mining work. However, exposure to natural sulfur in this area can cause health problems, especially in kidney function. This study aims to evaluate the relationship between exposure to toxic natural pollution and impaired kidney function. The study focuses on investigation of the toxic natural pollutants, namely SO_2_ and heavy metals in the air. Urine samples were collected from 60 respondents who were divided into two groups: 30 sulfur miners as the exposure group (cases) and 30 local residents (control) who were not engaged in mining activities. Urine samples were collected for creatinine level analysis, and glomerular filtration rate (GFR) was calculated to assess kidney function. In the case group, 66% (20 out of 30) had urinary creatinine levels above the normal limit (≥1.3 mg/dL), while in the control group, only 30% (9 out of 30) exceeded this value. GFR analysis showed that only two respondents (6.7%) in the case group had normal kidney function (GFR ≥90 mL/min), while 53.3% (16 respondents) were in the moderate kidney function decline category (GFR 30–59 mL/min) and 13.3% (4 respondents) had severe kidney function decline (GFR 15–29 mL/min). In contrast, the control group had eight respondents (26.7%) with normal kidney function as well as a lower prevalence of moderate (23.3%) and severe (3.3%) kidney function decline. Exposure to toxic natural pollution in Ijen Crater Volcano was significantly associated with an increased risk of kidney function impairment, as indicated by higher urinary creatinine levels and lower GFR values in the case group. These results emphasize the importance of health surveillance and implementation of preventive measures for mine workers in this biosphere reserve area.

## Highlights

• The Ijen Crater, a UNESCO biosphere reserve, is rich in sulfur and has the potential to disrupt kidney function.
• Clinical research involved 60 respondents: 30 sulfur miners and 30 residents for creatinine tests to assess kidney function.
• The urine creatinine in the traditional sulfur mining workers and residents have been analyzed and discussed.
• The Glomerular filtration rate (GFR) has been calculated, analyzed and discussed with the risk of kidney damage.

## Graphical Abstract

**Figure:**
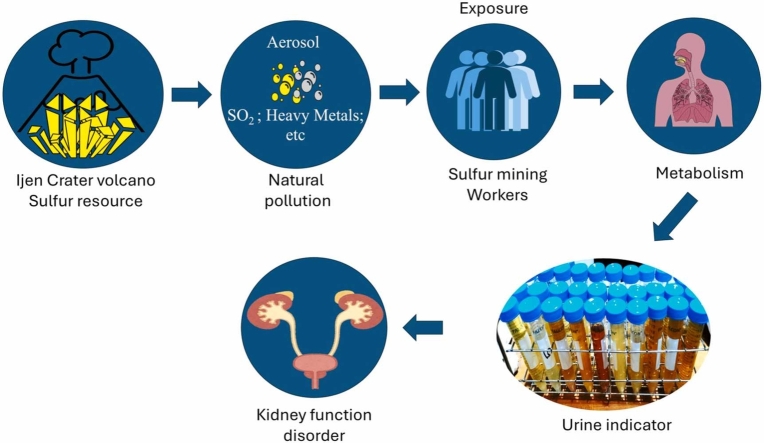

## 1 Introduction

Traditional sulfur mining in the Ijen Crater Volcano (ICV), East Java, is an integral part of the life of the local community and is one of the unique attractions in the world [1]. The ICV is famous for its "blue fire" phenomenon, a luminous blue flame only found in a few locations worldwide. In addition, the ICV has been recognized by UNESCO as part of the world's Biosphere Reserve, highlighting its biodiversity and ecosystem value [2]. However, behind its beauty the area holds major health challenges for the miners. Without adequate protection, miners are exposed to toxic gases such as sulfur dioxide (SO₂), hydrogen sulfide (H₂S), and heavy metals such as chromium, arsenic, and cadmium brought to the surface through volcanic and geothermal activity [3], [4]. The combination of these natural pollutants has great potential to affect health.

Urinary creatinine is an important indicator of kidney function [5], [6]. Urinary creatinine levels provide an overview of the kidney's filtering capacity, which can be an early marker of impaired kidney function [7]. This urinary creatinine value is then used to calculate the Glomerular Filtration Rate (GFR), which is the main parameter in assessing the kidney's ability to filter blood [8], [9]. GFR helps identify the level of kidney function, which can then be classified into various stages, ranging from normal function to severe kidney impairment or even end-stage kidney failure [9], [10].

In the context of extreme environmental exposure at Ijen Crater Volcano (ICV), urinary creatinine and GFR measurements can be used to evaluate the impact of natural pollutants on the kidney health of traditional sulfur miners [11]. Chronic exposure to toxic gases and heavy metals in this environment is known to trigger oxidative stress and kidney tissue inflammation, increasing the risk of kidney damage [12], [13].

To date, no study has specifically examined the impact of kidney health on traditional sulfur miners in the ICV despite the significant exposure to volcanic pollution and heavy metals in the area. Therefore, this study aims to identify urinary creatinine levels and GFR as markers of kidney dysfunction risk in a population of traditional sulfur miners in the ICV area. The results of this study are expected to provide important scientific data to develop better preventive measures and occupational health policies for sulfur miners, as well as support improved work safety in the traditional mining sector in volcanic environments.

## 2 Method

### 2.1 Location study and toxic natural pollution source

This study was conducted at the sulfur source of the ICV in Banyuwangi Regency, East Java, Indonesia. The source of natural pollution focused on gas and heavy metals analysis. Gas SO_2_ was found based on previous research [14]. Heavy metals analysis in the air were conducted using high volume air sampler (HVAS) using polytetrafluoroethyene (PTFE) filter for 8 h in the main work location. Heavy metals analysis used inductively coupled plasma mass spectrometry (ICP-MS to measure V, Cr, Co, As, Cd, and Pb.

### 2.2 Respondents and urine sampling

This study was conducted using a cross-sectional method, using 60 urine sample respondents, consisting of 30 sulfur miners and 30 control samples. Respondents were interviewed directly before urine sampling to obtain relevant baseline information regarding their health conditions. The data collected included anthropometric characteristics and health symptoms experienced by workers during mining activities, as well as physical conditions and health complaints.

Urine samples of workers were taken based on inclusion criteria, namely workers aged 18 +, who have worked as sulfur miners for at least 1 year and who are not under medical care or taking medication [15]. Urine samples were taken and collected during the work day. Control urine samples were community respondents who were not directly related to sulfur mining activities. Control respondents were taken on the condition that the respondent's age was 18 + and was not taking medication or receiving medical care. Urine samples were taken using a urine container, put into a tube, and then stored in a cooling box until analysis was carried out in the laboratory after 48 h.

### 2.3 Urine creatinine analysis

Urine samples were analysed using spectrophotometry (SCILOGEX SCI UV1100) at a wavelength of 510 ± 10 nm to measure creatinine levels. This creatinine measurement used the Jaffe method, as described by Chromý et al. [16]. The Jaffe method is a standard method for measuring creatinine that relies on the reaction between creatinine and picric acid, producing a color that can be measured at a certain wavelength. In this analysis, the reagents used consisted of 25 mmol/L picric acid, 300 mmol/L at pH 12.5 phosphate buffer, and 2.0 g/L, w/v sodium dodecyl sulfate (SDS), which helped increase the stability of the mixture. These reagents were mixed in a 1:1 ratio to form a working reagent.

For the analysis procedure, the urine sample was first diluted 100 times and 1000 times to ensure that the creatinine level was within the device's detection range. From the diluted urine solution, 0.1 mL was taken and added to 1 mL of the prepared working reagent, then mixed evenly for ± 30 s. After mixing, this mixture was analyzed using spectrophotometry. Before the sample analysis was carried out, a standard curve of creatinine concentration was first made as a reference, with concentration values on the curve being 0.1, 0.5, 1.0, 1.5, and 2.0 mL/dL. Spectrophotometry was performed at a wavelength of 490 nm for all samples after the standard curve was made. This wavelength was chosen to ensure optimal detection of the reaction results between creatinine and the working reagent. By comparing the absorbance results of the sample to the standard curve, the creatinine levels in the urine samples can be calculated accurately.

### 2.4 Data analysis

Urine creatinine data analysis was done by comparing it to the standard creatinine value. This comparison aims to assess how far the creatinine value in the urine sample is above or below the established threshold. Furthermore, the data are analysed using a statistical test, namely the mean difference test, to measure significant differences between the two groups of variables being compared. This test helps determine whether there is a real difference in creatinine values between the groups studied.

### 2.5 Glomerular filtration rate (GFR)

The GFR calculation was carried out using the Cockcroft-Gault method, which estimates the GFR based on serum creatinine levels. This method considers several variables, such as age, weight, sex, and serum creatinine levels, thus providing a more specific estimate of an individual's kidney function. Creatinine, a waste product of muscle metabolism, was consistently used as the primary biomarker in this analysis, as blood creatinine levels typically increase with decreasing kidney function. The equation for calculating GFR using the Cockcroft-Gault method is shown in Eq. 1 [17].

(1) $$\mathrm{GFR}\;\left(\frac{\mathrm{mL}}{\min}\right)=\frac{\left(140\;-\mathrm{age}\right)\;x\;\mathrm{weigh}\;\left(\mathrm{kg}\right)\;x\;(0.85\;\mathrm{for}\;\mathrm{female})}{72\;x\;\mathrm{Creatinine}\;\mathrm{serum}\;(\frac{\mathrm{mg}}{\mathrm{dL}})}\;$$

The calculated LFG value is then categorized as follows:

GFR ≥ 90 mL/min/1.73 m^2^ is called stage 1 (normal),

GFR 60–89 mL/min/1.73m^2^ is called stage 2 (mild kidney function decline),

GFR 30–59 mL/min/1.73m^2^ is called stage 3 (moderate kidney function decline),

GFR 15–29 mL/min/1.73m^2^ is called stage 4 (severe kidney function decline)

GFR < 15 mL/min/1.73m^2^ is called stage 5 or End-Stage Renal Disease.

### 2.6 Ethical approval

The sampling process followed ethical research rules. Before sampling, each worker was asked in an interview to make sure they joined the study voluntarily and without any pressure. All respondents received clear information about the type of study, its purpose, and the methods used. Only those who agreed voluntarily and signed the consent form were included in the study. This study met the applicable ethical guidelines and was approved by The Research Ethics Committee of Bandung Institute of Technology (ITB) with Protocol No. KEP/II/2024/X/M200224SHS-SOOH.

## 3 Results and discussion

### 3.1 Heavy metals and SO_2_ pollution

Table 1 shows the concentration values of heavy metals and sulfur dioxide (SO₂) levels at the research location. Based on the data in the Table 1, all detected heavy metals—vanadium (V), chromium (Cr), cobalt (Co), arsenic (As), cadmium (Cd), and lead (Pb)—showed concentrations exceeding the established threshold values. Cd was recorded at 2.66 ppb, with a threshold of 0.0022 ppb, or approximately 1209 times higher. Similarly, As and Pb were detected at approximately 339 and 400 times higher than safe limits, respectively. Exposure to heavy metals at this level poses a significant risk to human health [40], [41]; this is because all heavy metals except V are classified as carcinogenic and can cause disorders in vital organs such as the kidneys, liver, and nervous system.

**Table 1:** Heavy metals and SO_2_ concentration.

Content	V	Cr	Co	As	Cd	Pb
Heavy metals (ppb)	0.29	9.05	0.87	1.12	2.66	2.36
Threshold level value (ppb)*	0.024	0.235	0.008	0.0033	0.0022	0.0059

SO_2_ Sampling pointSO_2_ (mg/m^3^)	1	2	3	4	5	6
	3.14	3.46	3.62	6.29	18.24	16.98
Standard Value (mg/m^3^)*	0.65					

* TLV-TWA ACGIH standard

Furthermore, SO₂ concentrations at the six sampling points also showed very high values, ranging from 3.14 to 18.24 mg/m³ . All of these values far exceed the recommended air quality standard value, which is 0.65 mg/m³ . The points with the highest concentrations were recorded at points 5 and 6, respectively at 18.24 mg/m³ and 16.98 mg/m³ , or around 26–28 times higher than the safe limit. This condition indicates the presence of a strong source of SO₂ emissions, such as active volcanic activity or intensive combustion of fossil fuels. Overall, the high concentrations of heavy metals and SO₂ gas in this area pose serious environmental and health risks. These risks include increased potential for respiratory diseases, organ damage, and kidney disorders.

### 3.2 Respondent characteristics

Table 2 shows the characteristics of the respondents of study, which involved 60 respondents who were evenly divided between the sulfur mine workers group and the control group from the surrounding community. Most of the workers were over 40 years old (22 people), in contrast to the control group, which was mostly under 40 years old (16 people). The distribution of body weight showed that workers tended to weigh between 56–65 kg (14 people), while the control group was mostly in the > 65 kg category (14 people). The level of education also showed significant differences, where the majority of workers only completed elementary school (10 people) or junior high school (14 people), while the control group mostly had high school education (14 people) and college (4 people).

**Table 2:** Respondents’ characteristics.

Characteristic	Worker	Control
Total respondent (n)	30	30
Age		
Under 40 years old	8	16
Upper 40 years old	22	14
Weight (kg)		
< 50	0	2
50 – 55	5	4
56 – 65	14	10
> 65	11	14
Education level		
Elementary school	10	2
Junior high school	14	10
Senior high school	8	14
Higer university	0	4

The differences in characteristics between the worker and control groups, such as age, weight, and education level, reflect the conditions of the mine worker population and the general public. Despite the differences in characteristics, the control group still provides a valid comparison because they come from the same community and have similar environmental exposures, except for specific factors related to mining work [18], [19]. Therefore, this study can effectively evaluate the unique impacts of sulfur and heavy metal pollution exposure on the kidney health of mine workers, using the control group as a baseline for objective and scientific interpretation of the results.

### 3.3 Urine creatinine concentration

**Fig. 1:**
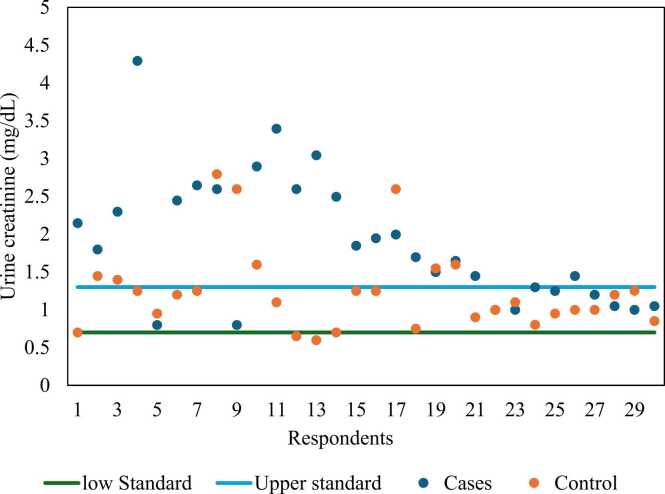
Urine creatinine concentration.

Urine creatinine data include measurements in the "Cases" and "Control" groups, with a standard range set between 0.7 and 1.3 mg/dL [20], [21]. Fig. 1 shows the value of urine creatinine for each respondent. The graph shows the distribution of urine creatinine levels (mg/dL) across all study respondents, consisting of cases (blue dots) and controls (orange dots). The horizontal axis indicates the respondent number, while the vertical axis indicates the urine creatinine level in mg/dL. In the graph, the green line represents the minimum standard for normal urine creatinine levels (0.7 mg/dL), while the light blue line represents the maximum standard or upper limit of normal (1.3 mg/dL). These two lines indicate whether each respondent's creatinine level is below the minimum standard, within the normal range, or above the upper limit of normal. There is a significant variation between the values in the "Cases" and "Control" groups, where many samples in the "Cases" group have creatinine concentrations above the standard range set. The majority, 20 out of 30 respondents (66%), have urine creatinine values above the standard (1.3 mg/dL). In contrast, in the "Control" group, most creatinine values are in the normal range of 0.7–1.3 mg/dL, with only 9 out of 30 respondents (30%) showing values that exceed the upper limit.

Urinary creatinine concentration provides important indications of a person's health condition, especially kidney function, hydration status, and physical activity [22]. When the creatinine concentration in urine is below normal standards, it can indicate impaired kidney function. In this case, the kidneys cannot effectively filter creatinine from the blood, which causes less creatinine to be excreted through urine. In addition, low creatinine can also occur in individuals with overhydration, where urine volume increases and causes creatinine concentrations to become more dilute [22]. Other conditions, such as low muscle mass, can also result in lower creatinine levels because the body produces less creatinine.

Conversely, higher than normal creatinine concentrations indicate dehydration [23]. When the body is dehydrated, the kidneys excrete less urine, which causes the concentration of substances in the urine, including creatinine, to increase [24]. This dehydration can occur in hot work environments, where the body loses more fluids. This increased urinary creatinine can also indicate that the kidneys are working harder to regulate the body's fluid balance.

In addition to dehydration, vigorous physical activity can increase urinary creatinine levels. Creatinine is a byproduct of the breakdown of creatine phosphate in muscles, so higher muscle activity, such as that experienced by sulfur miners, will increase creatinine production. In this condition, increased urinary creatinine levels do not necessarily indicate kidney problems but rather a normal body response to increased muscle metabolism. However, increased creatinine must also be monitored to ensure the kidneys are still functioning properly.

Thus, low and high creatinine concentrations differ greatly regarding the causative factors. Low creatinine values may indicate kidney disorders, overhydration, or low muscle mass, while high creatinine values are more often associated with dehydration, vigorous physical activity, or, in some cases, acute kidney injury. Additional data such as creatinine levels, hydration history, and the individual's history of physical activity or kidney health are needed to obtain a more accurate conclusion.

### 3.4 Statistical analyses

Table 3 shows the statistical analysis to determine the differences in variable values between case and control respondents. Based on the results of the Shapiro-Wilk normality test, the urine creatinine concentration data for the case group showed a distribution that was close to normal (p-value = 0.055), while for the control group, the data were not normally distributed (p-value = 0.0001). Because the data in the control group were not normally distributed, the assumption of normality was not met. Therefore, a non-parametric test such as the Mann-Whitney U Test was used to compare the two groups. The results of the Mann-Whitney U Test showed a U value of 668.5 with a p-value of 0.0013. This indicates a statistically significant difference between urine creatinine concentrations in the case and control groups. With a p-value less than 0.05, it can be concluded that there is a significant difference in urine creatinine concentration between the groups studied, where the urine creatinine concentration in the case group was significantly different compared to the control group.

**Table 3:** Statistical analyses.

Statistic test	Case	Control
normality test (Shapiro-Wilk)	p-value	
	0.055	0.0001
Mann-Whitney U Test	p-value	
	0.0013	

**Fig. 2:**
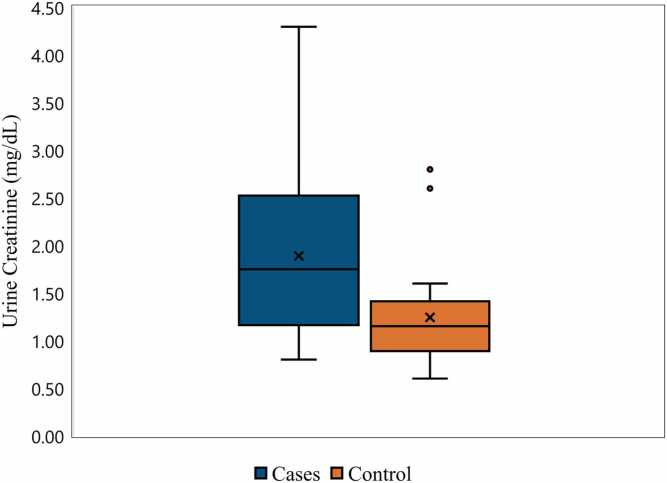
Boxplot of urine creatinine concentration on the cases and control.

Fig. 2 shows the Boxplot of urine creatinine between cases and controls. The boxplot shows a higher median urinary creatinine of about 1.75 mg/dL compared to the control group, with a median of about 1.0 mg/dL. In addition, the variability of creatinine levels in the case group was greater, with an interquartile range (IQR) of 1.2–2.6 mg/dL, compared to the control group, which ranged from 0.85 to 1.35 mg/dL. The case group's wider minimum and maximum values indicate a more dispersed data distribution.

The difference in creatinine levels between the two groups may reflect significant changes in kidney function in the case group. Increased creatinine levels can indicate kidney disorders, potentially caused by exposure to risk factors such as environmental pollution, chemical exposure, or certain health conditions [25], [26]. Meanwhile, in the control group, creatinine levels tended to be more uniform with lower variability, although two outliers indicated individuals with higher-than-normal urinary creatinine levels.

These differences in urinary creatinine distribution are important to note in the context of environmental health or occupational safety research. Elevated creatinine levels in the case group may indicate more serious health outcomes from exposure to environmental or occupational risks, such as exposure to toxic chemicals. However, further statistical analysis is needed to confirm this association and determine whether the visually apparent differences are statistically significant [27]. This will help draw stronger conclusions about the potential effects of the risk factors studied.

### 3.5 Glomerular filtration rate (GFR)

**Fig. 3:**
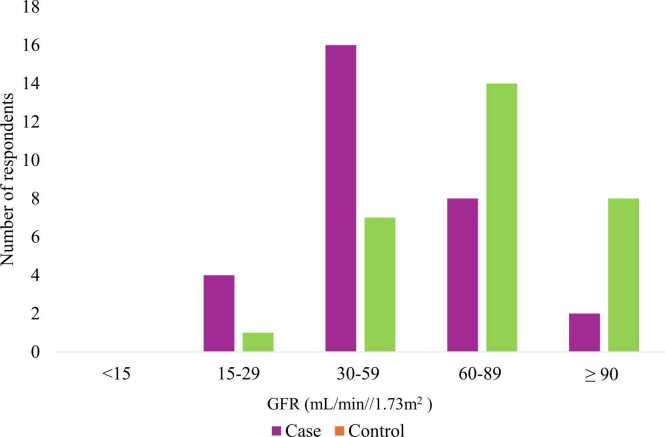
The GFR value of case and control respondents.

Fig. 3 shows the distribution of the number of samples in the case and control groups based on the GFR used to assess kidney function. GFR with a value of ≥ 90 mL/min is categorized as stage 1, indicating normal kidney function. In this Figure only two samples from the case group were in stage 1, compared to 8 samples from the control group. This indicates that individuals in the control group were more likely to have normal kidney function than the case group.

Mild kidney function decline (stage 2, GFR 60–89 mL/min) was also more common in the control group, with 14 samples, compared to 8 samples from the case group. However, more severe kidney function decline (stage 3, GFR 30–59 mL/min) was more dominant in the case group, with 16 samples compared to only seven samples in the control group. This indicates that the case group has a higher prevalence of moderate kidney function decline.

In stage 4, which indicates severe renal function decline (GFR 15–29 mL/min), there were four samples from the case group and only 1 sample from the control group. There were no samples in stage 5 (GFR <15 mL/min), indicating no cases of End-Stage Renal Disease (ESRD) in this group. Exposure to SO₂ through inhalation affects the renal Glomerular Filtration Rate (GFR) through molecular mechanisms primarily involving systemic oxidative stress and inflammation. When inhaled, SO₂ reacts with water in the respiratory tract to form sulfurous acid (H₂SO₃), which promotes the generation of reactive oxygen species (ROS), including superoxide (O₂⁻) and hydrogen peroxide (H₂O₂). These oxidative molecules can spread systemically through the bloodstream and induce renal oxidative stress, damaging the glomerular endothelium through lipid peroxidation in podocytes and mesangial cells, decreasing nephrin expression in the slit diaphragm, and ultimately impairing glomerular filtration. Simultaneously, SO₂ exposure stimulates alveolar macrophages to release pro-inflammatory cytokines such as Tumor Necrosis Factor-Alpha (TNF-α) and Interleukin−6 (IL−6), which may reach the kidneys and activate inflammatory signaling pathways, particularly Nuclear Factor Kappa B (NF-κB) and NLR Family Pyrin Domain Containing 3 (NLRP3) inflammasome activation, thereby amplifying local inflammation and oxidative injury. Persistent oxidative stress may further activate NOX4 (NADPH oxidase 4), a major intrarenal source of ROS, and trigger the Transforming Growth Factor-Beta/Smad (TGF-β/Smad) pathway, promoting extracellular matrix accumulation, tubulointerstitial fibrosis, and glomerulosclerosis. In addition, dysregulation of nitric oxide (NO) and endothelin−1 may induce afferent arteriole vasoconstriction, reducing renal perfusion and filtration pressure. Collectively, these interconnected molecular mechanisms progressively reduce functional nephron integrity and glomerular filtration surface area, ultimately contributing to the decline in GFR observed among traditional sulfur miners chronically exposed to volcanic sulfur gases [42], [43], [44]. Epidemiological evidence from studies in industrial areas shows that chronic exposure above 0.21 mg/m³ is correlated with decreased GFR, especially in workers with increased systemic risk after a 30-year projection

This study showed differences in the distribution of kidney function between the case and control groups based on GFR values. The control group had better overall kidney function than the case group, where there was a higher prevalence of moderate to severe kidney function decline (stages 3 and 4). These data indicate the possibility of an impact of work or environmental exposure on kidney function in the case group.

Table 4 shows that of the GFR data collected from multiple studies provide important insights into how environmental exposures and specific conditions affect kidney function in different populations. In this study, sulfur mine workers showed an average GFR of 30–50 mL/min, reflecting moderate to severe kidney function impairment. In contrast, the control group had an average GFR of 60–89 mL/min, indicating mild kidney function impairment. These results support the idea that chronic exposure to sulfur and natural pollutants around mining sites can worsen kidney health.

**Table 4:** Comparison to GFR values in other studies.

No	Case	Type of respondent	Insight GFR value	Source
1	Natural pollution in the sulfur mining	Total 60 respondent with 30 Worker sulfur mining and 30 control	1. Worker: mean GFR of worker was 30–50 mL/min. 2. Control: mean GFR value was 60–89 mL/min.	This study
2	General participant in the hospital	Patients	1. Mean GFR of the overall patient group was 64.1 ± 33.5 mL/min. 2. CONTROL group had a GFR of 96.6 ± 22.4 mL/min (n = 193).	[28]
3	Environmental pollution (heavy metals and air pollution)	Population community	GFR < 60 mL/min/1.73 m².	[29]
4	Patients typically present with symptoms such as fatigue, swelling,	patients with chronic kidney disease	GFR 53.0 mL/min/1.73 m²	[30]
5	General population	Pakistani community	47% of participants had an eGFR of ≥ 90 mL/min per 1.73 m²	[31]
6	Airborne pollution (PM, and heavy metals)	Residents	The study reported a median eGFR value of 60 mL/min/1.73 m² in participants with chronic kidney disease (CKD) at baseline.	[32]
7	Each patient's exposure to ambient air pollutants	patients diagnosed with chronic kidney disease	It mentions that renal progression is considered when estimated glomerular filtration rate (eGFR) decreases more than 25% from the baseline eGFR.	[33]
8	The study specifically looked at the relationship between environmental lead exposure	The research involved 8592 participants from the Prevention of Renal and Vascular Endstage Disease (PREVEND) study,	higher blood levels of lead were associated with lower eGFR levels and impaired kidney function (eGFR < 60 mL/min/1.73m) in 3941 US adults.	[34]
9	The study investigated the impact of long-term exposure to ambient air pollutants, specifically particulate matter (PM_2.5_ and PM_10_) and nitrogen oxides (NOx).	population-based cohort from the Swedish CArdioPulmonary Study (SCAPIS), which included middle-aged men and women from six Swedish cities	The study found a trend towards a negative association between two-year average exposure to nitrogen oxide (NOx) and estimated glomerular filtration rate (eGFR), indicating a potential decrease of 0.29% in eGFR per 10 µg/m^3^ higher NOx exposure.	[35]
10	inhalable particles (PM_10_) and sulfur dioxide (SO_2_), was associated with lower eGFR values in adolescents.	The study analyzed data from the 'Children of 1997' Hong Kong birth cohort, which included a total of 8327 participants.	The study found that exposure to PM_10_ and SO_2_ during early life, particularly in utero, was associated with reduced estimated glomerular filtration rate (eGFR) in adolescents, indicating impaired kidney function linked to air pollution exposure.	[36]
11	investigated the association between long-term ambient air pollution exposure and renal health	The respondents included 10,942 children and adolescents aged 25 years or younger from Taiwan and Hong Kong, observed from 2000 to 2017	Each 10 µg/m^3^ increase in PM_2.5_ was associated with a 0.45 µL/min/1.73 m² reduction in the yearly increase in estimated glomerular filtration rate (eGFR), indicating that pollution exposure negatively impacts renal function in children and adolescents.	[37]
12	Exposure of air pollution, specifically CO, NO, NOx, and PM_2.5_	The study enrolled 447 respondents who were patients with chronic kidney disease (CKD) from the Pingtung Hospital	The study found that increased concentrations of air pollutants, specifically CO, NO, NOx, and PM_2.5_, were significantly associated with long-term deterioration in estimated glomerular filtration rate (eGFR) in chronic kidney disease patients, The estimated glomerular filtration rate (eGFR) for the study population decreased by 1.9 mL/min/1.73m² per year.	[38]

Support for these findings can be found in other studies. Hui-Ju et al. [29] reported that populations exposed to heavy pollution had GFRs below 60 mL/min/1.73 m², confirming the negative impact of environmental pollutants on kidney function. Furthermore, Chung et al. [38] found that long-term exposure to air pollutants such as CO, NO, NOx, and PM_2.5_ caused an annual decrease in GFR of 1.9 mL/min/1.73 m² in patients with chronic kidney disease (CKD). This finding is consistent with the results of a group of mine workers, where long-term exposure to natural sulfur pollution may have a similar effect.

In addition, air pollution also affects young age groups. Shi et al. [36] reported that inhalation exposure to particulate matter (PM_10_) and sulfur dioxide (SO_2_) during prenatal and early life was correlated with decreased GFR in adolescents. Meanwhile, Guo et al. [37] found that an increase of 10 µg/m³ PM2.5 caused an annual decrease in GFR of 0.45 mL/min/1.73 m² in children and adolescents. These exposures demonstrate the cumulative impact of air pollution on kidney function across life stages, even in individuals not directly exposed to specific pollution sources such as sulfur mines.

From a global perspective, Diana et al. [34] also showed that higher blood lead levels were correlated with lower eGFR, based on data from 8592 participants in the PREVEND study. This study and other data, such as those reported by Satarug et al. [39], who found that 6.9% of subjects exposed to cadmium pollution had a GFR below 60 mL/min, reinforce that exposure to heavy metals and air pollutants has a universal impact on kidney function, regardless of geographic location.

In conclusion, these results show a clear pattern that exposure to environmental pollutants, both natural and anthropogenic, significantly affects kidney function. This study, focusing on sulfur exposure from natural mines, contributes to understanding the relationship between environmental factors and kidney function decline and highlights the need for preventive measures to protect vulnerable populations.

### 3.6 Limitation of research

One limitation of this study is the lack of control over the workers' dietary intake during the study period. Kidney dysfunction, including changes in creatinine levels, is not only influenced by SO₂ exposure but can also be influenced by daily lifestyle factors, such as diet, fluid intake, smoking habits, alcohol consumption, and other individual health conditions. Therefore, the results obtained in this study cannot be fully attributed solely to SO₂ exposure in the workplace. Future research is recommended to consider a more comprehensive assessment of lifestyle and nutritional status, as well as longitudinal monitoring, to gain a deeper understanding of the combined influence of environmental exposures and lifestyle factors on kidney function in workers.

## 4 Conclusion

This study shows that exposure to natural sulfur in ICV significantly affects the kidney function of sulfur miners compared to local residents who are not directly exposed. Urinary creatinine analysis showed that 66% of miners (20 out of 30 respondents) had creatinine levels above the normal limit (>1.3 mg/dL), while only 30% of respondents from the control group (9 out of 30) showed similar results. This reflects the higher level of sulfur exposure in the miner group, which is at risk of causing kidney disorders. The results of GFR measurements also showed striking differences between the two groups. In the miner group, only 2 out of 30 respondents had GFR ≥ 90 mL/min (normal kidney function category), while the majority were in the category of decreased kidney function, namely GFR 30–59 mL/min (16 respondents) and GFR 15–29 mL/min (4 respondents). In contrast, in the control group, eight respondents had GFR ≥ 90 mL/min, and most were at GFR 60–89 mL/min (14 respondents), reflecting relatively better kidney function. These results highlight the significant health risks of exposure to natural sulfur pollution in the ICV. Miners who are directly exposed have a higher prevalence of impaired kidney function, as reflected by high urinary creatinine levels and decreased GFR values. These findings emphasize the need for mitigation measures such as routine health surveillance, personal protective equipment provision, and sulfur exposure reduction through environmental approaches and occupational health policies.

## Authors contribution

Septian Hadi Susetyo (Conceptualization Sampling, Method, analysis, writing draft and revision); Azham Umar Abidin (Writing draft, revision manuscript); Taiki Nagaya (draft review, evaluation, revision manuscript); Nobuyuki Kato (draft review, evaluation, revision manuscript); Emi Fukuda (draft review, evaluation, revision manuscript); Yasuto Matsui (Supervisor, validation, draft review, revision manuscript).

## Funding

There is no funding in this research.

## Declaration of Competing Interest

The authors declare that they have no known competing financial interests or personal relationships that could have appeared to influence the work reported in this paper.

## Data Availability

The datasets generated and/or analyzed during the current study are available from the corresponding author on reasonable request.
